# Challenging cases during clinical clerkships beyond the domain of the “medical expert”: an analysis of students' case vignettes

**DOI:** 10.3205/zma001238

**Published:** 2019-05-16

**Authors:** Patrik Bächli, Claudine Meindl-Fridez, Anja Nikola Weiss-Breckwoldt, Jan Breckwoldt

**Affiliations:** 1 Kantonsspital Aarau, Dept. of Anesthesiology, Aarau, Switzerland; 2 University Hospital Zurich, Department of General Internal Medicine, Zurich, Switzerland; 3 Ambulatorium Römerhof, Zurich, Switzerland; 4 University Hospital Zurich, Institute of Anesthesiology, Zurich, Switzerland

**Keywords:** workplace based training, hidden curriculum, CanMEDS roles, clinical clerkship, clinical case vignettes

## Abstract

**Background:** During clinical clerkships students experience complex and challenging clinical situations related to problems beyond the domain of the “Medical Expert”. Workplace routine may leave little opportunity to reflect on these situations. The University of Zurich introduced a mandatory course directly after the clinical clerkship year (CCY) to work up these situations. Prior to the course each student submitted a vignette on a case he or she had perceived challenging during the CCY and which was not related to the domain of the “Medical Expert” role. In this paper we want to characterize these cases in respect to most prominent themes and related CanMEDS roles. The goal was to inform clinical supervisors about potential teaching demands during the CCY.

**Methods:** All case vignettes submitted by a years’ cohort were analysed by three researchers in two ways:

1. for the clinical characteristics and the main theme of the underlying problem and
2. the most prominent CanMEDS roles involved.

Themes of the underlying problem were aggregated to overarching topics and subsequently to main categories by pragmatic thematic analysis.

**Results: **254 case vignettes covered the whole spectrum of clinical disciplines. A wide range of underlying themes could be assigned to five main categories: “communication within team” (23.2%), “communication with patients and relatives” (24.8%), “patient behavior and attitudes” (18.5%), “clinical decision making” (24.0%), and “social and legal issues” (9.4%). Most frequent CanMEDS roles were “Communicator” (26.9%) and “Professional” (23.5%).

**Conclusions:** Cases students perceived as challenging beyond the “Medical Expert” were reported from all clinical disciplines. These were mainly related to communicational and professional issues, mirrored by the CanMEDS roles “Communicator” and “Professional”. Therefore, supervisors in clinical clerkships should put an additional teaching focus on communication and professionalism.

## Zusammenfassung

**Hintergrund: **Während des Praktischen Jahrs (in der Schweiz: Wahlstudienjahr, WSJ) werden die Studierenden mit komplexen Problemen konfrontiert, die oft nicht allein mit Mitteln im Bereich der CanMEDS-Rolle „Medical Expert“ gelöst werden können. Während der Arbeit im Spital bleibt meist wenig Zeit diese Situationen aufzuarbeiten. Die Universität Zürich hat daher einen Pflichtkurs eingeführt, welcher den Studierenden direkt nach dem Wahlstudienjahr Raum zur Reflexion gibt. Als Kursgrundlage reichen alle Studierenden eine Vignette zu einem klinischen Fall ein, den sie im WSJ als besonders herausfordernd erlebt haben und dessen Problem ausserhalb des Bereichs des „Medical Experts“ lag. Diese Arbeit untersucht, welche Themenbereiche in diesen Fallvignetten am häufigsten angesprochen wurden und im Bereich welcher CanMEDS-Rollen die Problematik angesiedelt war. Die Ergebnisse sollen klinischen Betreuenden helfen, mögliche herausfordernde Situationen zu erkennen und diese mit den Studierenden zu thematisieren.

**Methoden: **Drei klinisch erfahrene Ärzte analysierten sämtliche Fallvignetten eines Studienjahres aus zwei Perspektiven:

1. bezüglich der Hauptthematik des zugrundeliegenden Problems und
2. bezüglich der hauptsächlich betroffenen CanMEDS-Rolle.

Mittels induktiver Verfahren wurden die Hauptthematiken zu übergeordneten Kategorien zusammengefasst.

**Resultate: **254 eingereichte Fälle verteilten sich über sämtliche medizinische Fachgebiete. Die Hauptthematiken konnten fünf übergeordneten Kategorien zugewiesen werden: „Kommunikation im Team“ (23.2%), „Kommunikation mit Patienten und Angehörigen“ (24.8%), „Verhalten und Haltungen der Patienten“ (18.5%), „ärztliche Entscheidungsfindung“ (24.0%) und „Soziale und rechtliche Probleme“ (9.4%). Die am häufigsten vertretenen CanMEDS-Rollen waren „Communicator“ (26.9%) und „Professional“ (23.5%).

**Fazit:** Die Vignetten zu herausfordernden klinischen Fällen stammten aus sämtlichen medizinischen Fachgebieten. Am häufigsten wurden Probleme in den Bereichen „Kommunikation“ und „Professionelles Handeln“ identifiziert. Dies spiegelt sich wieder in den am häufigsten vertretenen CanMEDS-Rollen „Communicator“ und „Professional“. Diese Befunde sollten betreuende Ärztinnen und Ärzte dafür sensibilisieren, dass Studierende im Wahlstudienjahr herausfordernde Situationen in diesen Bereichen erleben. Die Thematisierung im klinischen Kontext erscheint daher sinnvoll.

## Background

Working experience in routine clinical settings is essential for undergraduate medical education [1], [2], [3]. An important facet of this learning environment is related to non-standardized situations, where plain clinical algorithms may not be sufficient to solve the problem [4]. These complex clinical situations often originate from causes beyond the domain of the CanMEDS role “Medical Expert” [5], e.g. from communication and teamwork problems [6], resource shortages, or ethical dilemmas [7], [8]. During clinical clerkships students may struggle with the less formal way of learning in the clinical workplace [9], [10], [11], [12]. As a challenge for teaching, there may be limited time to think through and discuss such complex situations in appropriate depth. Since this area of training is difficult to capture by specific learning objectives, it may well slip away into neglected and informal parts of the curriculum, often referred to as “hidden” [13], [14], [15]. As a consequence, valuable teaching opportunities are likely to be missed. Hence, there clearly is a need to better understand the circumstances of cases which students perceive as challenging. With this knowledge supervisors could better tailor clinical teaching to issues, which are related to other CanMEDS roles than the “Medical Expert” and which may be part of the “hidden curriculum”.

At the University of Zurich the clinical situations mentioned above are addressed by a mandatory course located directly after the CCY. For this course each student has to hand in a written case vignette of a challenging clinical situation which relates to a problem beyond plain medical content. We sought to analyze these vignettes from two perspectives:

1. by retrieving the clinical characteristics and the main underlying themes of the cases, to capture the perspective of clinical supervisors (inductive approach) and
2. identifying the most prominent CanMEDS roles within the case in order to link the content to the framework of the Swiss national catalogue of learning objectives, which in part is based on the CanMEDS framework [16] (deductive approach).

In combining the two strategies we assumed to arrive at more consolidated results. Final goal was to provide information to CCY supervisors in order to better tailor teaching in the workplace to the students’ demands.

## Methods

### Curricular context

In German-speaking countries, university studies in medicine take six years. The curriculum at the University of Zurich is outcome-based and horizontally integrated in all study years, incorporating additional elements of vertical integration. The first two years focus on basic science and basic clinical topics. Third and fourth year provide a comprehensive and systematic approach to clinical medicine, finishing with an 8-station Objective Structured Clinical Examination (OSCE). During these two years students visit patients in standardized bedside teaching courses facilitated by experienced physicians (“clinical courses”). Patients are preselected to best fit into the curricular context. Students are guided during their patient encounters and do not take decisions on their own. During the fifth year (the CCY) students exclusively learn in workplace settings in various clinical disciplines and institutions which they may choose freely [17], [18]. A logbook based on the Swiss Catalogue of Learning Objectives (SCLO) [16] provides guidance in terms of general skills, reflective practice and formative workplace based assessment (e.g. “Mini-CEX” [19]). Taking up experiences from the CCY, the sixth year is designed to focus on “problems as starting points for training”, as specified by the SCLO [16]. Finally, it prepares for the federal licensing examination and subsequent postgraduate training.

#### „Challenging clinical case“

In the term scheduled directly after the CCY, the University of Zurich provides a mandatory workshop-like course to reflect on physicians’ roles, partly drawing on the CanMEDS roles framework [5]. Prior to this course each student has to submit a “challenging clinical case” vignette of 150-300 words from his or her own CCY experience. The cases are requested to cover problems outside of the CanMEDS role “Medical Expert” and serve as a basis for discussion during the workshops (17-18 students) and in small group discussions (5-6 students). The “challenging clinical case” is defined in a broad fashion, including potential underlying causes in the fields of communication, resources, ethics, or decision making (for a translation of the students’ assignment see attachment 1 ).

#### Analysis of case vignettes

In the spring term 2015, electronic files of all “challenging clinical case” vignettes were used. Prior to analysis we anonymised any information, which could have identified specific persons or institutions. The aim of the analysis was to capture a broad and authentic clinical perspective of clinical supervisors. For rating, we therefore chose experienced clinicians from one major somatic non-surgical, one surgical-interventional, and one non-somatic discipline (in specific: general internal medicine, anaesthesiology and psychiatry). The raters were based in hospital settings reflecting that more than 95% of the CCY placements were in-hospital. The raters had substantial experience in clinical teaching, were closely familiar with the CanMEDS roles framework (which is well anchored in the Swiss undergraduate curricula by means of the SCLO) and were experienced in clinical curriculum planning. In view of the inherent overlap between some CanMEDS roles [20], [21], [22], [23] in combination with our purpose of researcher triangulation no further rater training was undertaken. The raters independently assessed all case vignettes for patient gender and age, patient’s disease or condition, primary clinical discipline involved, the prominent underlying theme of the reported problem, and CanMEDS roles. The prominent underlying theme was provided as a keyword, or a short phrase (e.g. “hidden agenda of patient, legal issue”). For analysis in respect to CanMEDS roles the three most appropriate roles were selected by order of importance. It was possible to choose less than three roles (matrix see attachment 2 ). 

#### Further data analysis

Results were filled into a spreadsheet file and further analysed by descriptive statistics. For analysis of the underlying themes, we compared the results of the raters for concordance. In case of dis-concordance a decision was made by a fourth member of the research team (PB) based on raters’ decisions and a review of the case vignette. From the set of resulting themes two researchers (PB and JB) aggregated the themes to over-arching topics using an abbreviated pragmatic approach of thematic analysis [24]. In a second step these topics were assigned to potential main categories, reviewed, discussed and revised to be finally grouped to main categories [24]. 

For analysis of the frequencies of CanMEDS roles the raters’ decisions were combined. We weighed the roles of first, second and third priority in a 3:2:1 ratio. To avoid an artificial increase of statistical sample size we multiplied all roles named as first priority by 3/6, those named second by 2/6 and roles named third by 1/6. From these data we calculated the relative overall frequency of CanMEDS roles. In addition, we analysed the material for frequent combinations of CanMEDS roles. 

Finally, we compared the frequency of assigned CanMEDS roles between the raters. Given the obvious overlap between some of the roles as well as the raters’ backgrounds in different clinical disciplines, appropriate statistical comparison appeared difficult. Furthermore, the appropriate use of Cohen's kappa is limited to the comparison of two raters [25]. In agreement with the Institute of Epidemiology, Biostatistics and Prevention of the University of Zurich we therefore made an a priori definition of “very good” agreement, if all three raters agreed in two or more of the three roles of a case, irrespective of the priorities. If one of the roles was agreed on by all three raters, we classified this as “acceptable agreement”. 

For statistical analysis the program “R” was used (version 3.2.2) in combination with “RStudio” (version 0.99.486), both open source, available from: https://www.r-project.org/ and https://www.rstudio.com.

#### Data safety and ethics

All data analysed were part of the routine course material students had handed in. Any information, which could have identified individuals or institutions was anonymized prior to analysis. According to the ethical committee of the Canton of Zurich formal consent by the students was not necessary. The committee stated no objections to the study (No. 099-2015).

## Results

### Student characteristics

From the total cohort of 256 students, 254 (99.2%) “challenging clinical cases” could be analysed; two students never handed in their case. Ratio of female students to males was 53.9% to 46.1%, median age was 25 years (10^th^-90^th^ percentile: 23-30). 

#### Patients and types of diseases

Patients’ ages were well distributed over the whole span of life, median was 50 years (range: newborn to 102y, see attachment 3 ). Most cases were reported from internal medicine (31.0%), followed by surgery (15.9%) and paediatrics (11.1%). Independent from the primary medical discipline 31.9% of cases occurred in emergency settings, from the surgery cases this even made up for 60% (24/40). Further details are shown in table 1 (Tab. 1).

#### Most prominent underlying themes of cases

The material showed a wide distribution of underlying themes (see table 2 (Tab. 2)). Themes were aggregated to 29 over-arching topics which were then grouped to the five categories: “Communication within team”, “Communication with patients & relatives”, “Patient behavior and attitudes”, “Clinical decision making”, “Social and legal issues”. 

Themes relating to ***communication within teams*** (59 cases), included hierarchy conflicts and interprofessional as well as interdisciplinary communication. Typical examples were that students were appointed to perform tasks which they did not feel prepared for (e.g. deliver an unfavorable diagnosis), or when they felt divided between nursing staff and physicians in regard to treatment. One quotation was:

*“Often, the nursing staff was overburdened and called for pharmacologic immobilization [of patients]. The physician in charge however, did not see a medical indication. This led to strong tensions between physicians and nurses and culminated in an emergency meeting …”* [case 055].

The second overarching topic was ***communication with patients and relatives*** (63 cases). Difficulties in these cases related to shared decision making, dealing with aggressive patients, acceptance of diagnosis or diverging information between patients and relatives. Many cases involved more than one group of persons, e.g. if patients, their relatives, physicians and nursing staff were involved in a difficult treatment decision. A typical quotation was: 

*“It was a problem that on each occasion a different family member was present and that they all argued in different directions how to proceed [with the patient].”* [case 228].

For the third overarching topic ***“patient behavior and attitudes”*** (47 cases) we assigned cases if the underlying problem was primarily triggered by a patient, including issues of patients’ autonomy and self-determination (like refusal of treatment, adherence to medication, misconception of disease), which may be illustrated by the following:

*“The recommendation for therapy […] was primarily accepted, but on the following day – after having consulted a healer within the circle of friends – therapy was requested to be withheld. The healer proposed olibanum and myrrh.”* [case 011].

We identified 61 themes to be assigned to the fourth overarching topic ***clinical decision making and treatment***, including diagnostic and treatment errors, complex decision making and therapeutic failure. One example quotation says:

*“[…] this case was rather frustrating for all persons involved, as the patient’s situation had become worse - despite the fact that the most severe differential diagnoses had been ruled out.”* [case 073].

The final overarching topic was formed by various ***social and legal issues*** (24 cases) including child abuse, psychosocial problems or illegality with the main portion relating to resource shortages. An exemplary quotation was: 

*“[patient with acute recurrent upper intestinal bleeding:] We wanted to repeat gastroscopy to ligate the oesophageal varices again. However, ligatures had gone out of supply in the hospital.” *[case 194].

#### Most frequent CanMEDS roles 

**Figure 1:**
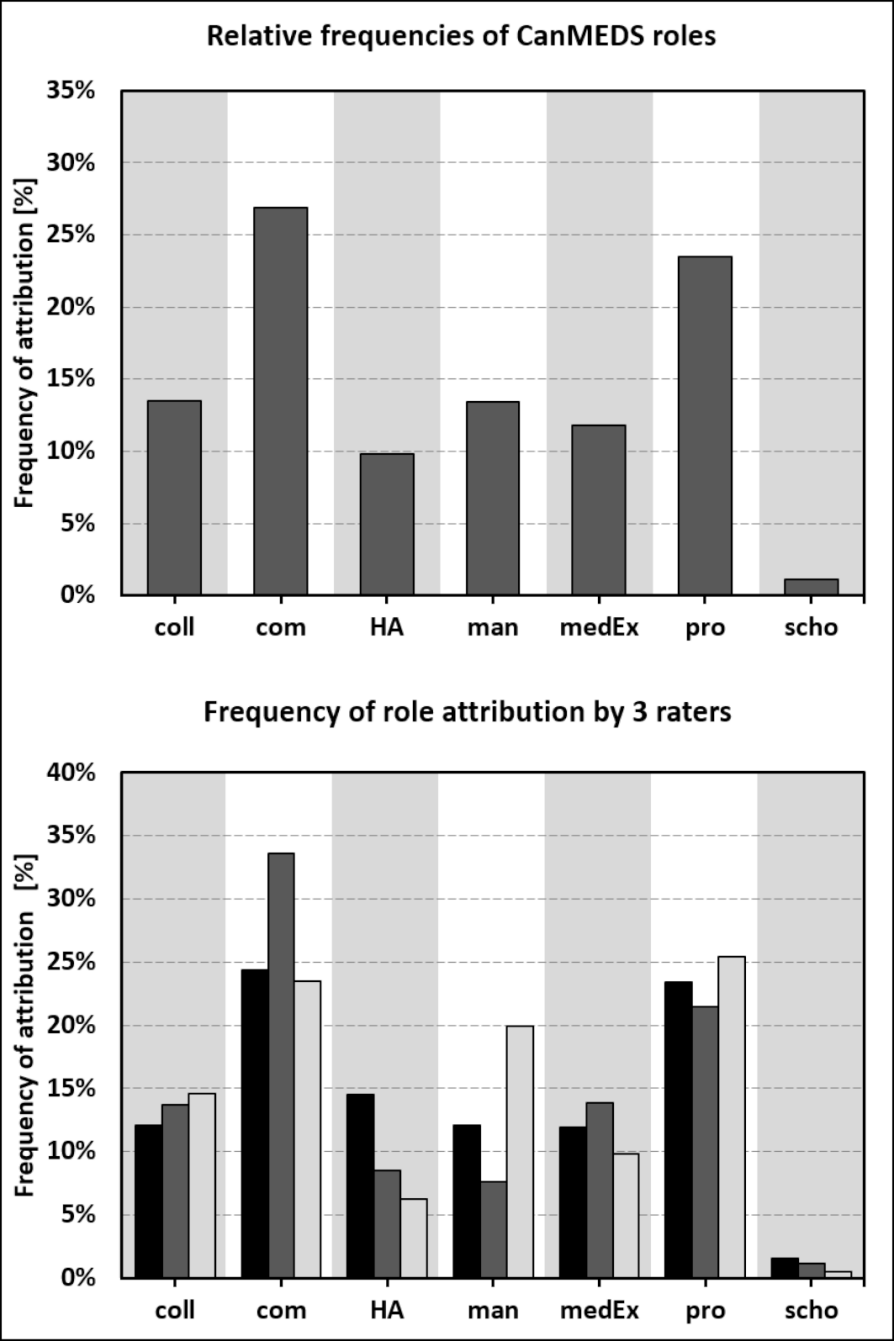
Frequencies of CanMEDS roles within “challenging clinical cases”, weighted according to priority. Top half of figure: mean of all three raters. Bottom half of figure: individual data from each rater (black: psychiatrist, dark grey: internist, light grey: anaesthetist). coll = Collaborator, com = Communicator, HA = Health Advocate, man = Manager, medEx = Medical Expert, pro = Professional, scho = Scholar

The “Communicator” and the “Professional” role were most frequently assigned (26.9% and 23.5 %); weighed overall frequencies are shown in figure 1 (Fig. 1) (upper part). The roles which raters selected with first priority showed a similar pattern (“Communicator” 31.3%, “Professional” 26.8%, see attachment 4 ). The two most frequent combinations of roles were “Communicator” & “Professional” and “Manager” & “Collaborator” (see table 2 (Tab. 2)).

#### Rater agreement for assessment of CanMEDS roles

In 200 cases all raters agreed at least on one role (78.7%) In specific, raters agreed on only one role in 139 cases (54.7%), on two roles in 58 cases (22.8%), and on all three roles in three cases (1.2%). This compares to a hypothetical random distribution of twelve cases with two agreements and zero cases with three agreements (calculated by program “R”). According to our predefinition, agreement was very good in 24.0% of the cases and acceptable in 54.7%. Weighted frequencies of roles as assessed by each of the three raters are depicted at the bottom part of figure 1 (Fig. 1). The roles on which raters agreed most often were “Communicator” and “Professional (108 and 68 cases, see attachment 5 ). 

## Discussion

“Challenging Clinical Case” vignettes provided a broad insight into what students found challenging beyond the “Medical Expert” role during their CCY. The two analytical approaches led to similar results. Most frequent underlying themes (inductive approach) were related to communicational problems in various combinations and to professionalism (clinical decision making, diagnostic error, treatment failure, psycho-social and legal problems). The (deductive) approach using the CanMEDS framework arrived at the “Communicator” and “Professional” as the most prominent roles, perfectly matching with the findings from the analysis of themes. In our view these findings reflect the domains which lack a formal representation within the curriculum. 

In respect to communicational issues many students, who worked as full members of a medical team for the first time in their lives, experienced the complexity of communication with patients, their relatives, colleagues, superiors and other healthcare professions (sometimes all at the same time). In contrast to the preceding study years of guided and selected patient encounters communication now became essential for students in order to get their work done, to keep one’s place within the team and to receive the required teaching [4]. Notably, students rather struggled with problems which were closer to their personal working situation than to issues in the domain of the “Collaborator” and “Manager” roles. The “Manager” role may have been less important because CCY students do not primarily act in this role while for the “Collaborator” role the students (being novices in the workplace) may not have perceived themselves as true collaborators. It may well be that the perspective would shift during postgraduate training [21].

The second overarching topic, professionalism, interrelates with the communicational challenges. Students were forced to reflect on their professional position when dealing with uncertainty or ambiguity [26], observing that decisions in clinical settings may not be clear cut by simply drawing on standard algorithms. This discrepancy may not be perceived by those team members who have already got familiar to such situations [27]. It should therefore be accounted for by supervisors and made explicit, that entering CCY constitutes a transition were perspectives change [28]. We find it important that these situations should be viewed as teaching opportunities rather than as threats [29], [30] turning a predominantly negative connotation of the “hidden curriculum” to a positive one [15]. Apparently, there is a need for appropriate teaching formats to address the “hidden curriculum” of clerkships, since the limitations students face often may not be discussed with a supervisor right away. Formal teaching provides an opportunity to use cognitive dissonances [31] as a motivator for reflection. The situated learning experience of the CCY could further be enriched if clinical supervisors encouraged their students to deliberately reflect on such situations and to integrate reflection into their clinical practice. One approach of our institution is to address these situations by a course on “challenging clinical cases” directly following the CCY. However, it may be more effective to discuss the cases while the clerkship is still going on by directing informal teaching towards a “supportive educational dialogue” [32], and by enhancing longitudinal educational relationships during clerkships [33]. Further, existing structured programs to support CCY ward round skills [34] could be enriched by reflective sessions, which may be inspired by “Balint group” elements [35], [36]. Another approach could be to prepare students more explicitly prior to the CCY to anticipate “challenging cases” [18], however, this would not substitute real-life experience.

The cases described occurred at any patient age and medical discipline, therefore the results are relevant to all clinical supervisors. The spectrum of clinical disciplines roughly represented the distribution of CCY stations students of our cohort had chosen [17]. Yet, working in emergency settings was associated with a higher incidence of “challenging cases”, especially in surgery placements. Possible explanations may be the time pressure inherent to emergencies, and that students in emergency departments often felt that they were given more temporary responsibility – which sometimes resulted in overburden. In addition, emergency settings are stressful for patients and their relatives with the potential to induce miscommunication [37]. This finding is highly important for clinical supervisors in emergency settings, especially as the need for work-up may easily get lost under time pressure.

Of interest is also, that students reported 25 cases from psychiatry (9.8%). When extrapolating results from the survey by Dupuis [17] 45 students from our cohort had completed a rotation in psychiatry. This means, that every second of these students chose a challenging case from psychiatry, despite having worked in other disciplines for much longer periods. Also of note may be, that eight cases (3.1%) related to anorexia nervosa patients, which is far more than would have been expected from natural prevalence [38]. This could be explained by the dynamics of this disease with a high potential for emotional involvement of students and complex communicational interactions between patients, relatives and health professions involved [39].

### Strengths and limitations 

We regard the high sample size and response rate as a strength of this study, providing a comprehensive picture of the student cohort. Secondly, an appropriate study setting was found regarding the time point of measurement and the task assigned to the students (construct of the “challenging clinical case”). This ensured emotional involvement of students and provided relevant data.

Rater agreement on themes of the cases was high between the raters and made final decisions easy. As anticipated, variation was higher for the analysis of CanMEDS roles, though data showed acceptable agreement according to our predefinition. However, we were unable to provide a positivist-quantitative statistical measure. Since this was a primarily qualitative study, we accepted this condition.

The study is limited by the setting at a single institution. However, curricula in Switzerland do not differ significantly as far as the CCY is concerned. Also, the hospitals and institutions our students chose for CCY represented the whole spectrum of workplace training. As an additional limitation social desirability bias may be discussed for the content of the case vignettes, however, no grades were given to the students. One could also argue in the opposite direction, that students might not have felt comfortable to share highly problematic cases, so that such cases would have been missed.

## Conclusions

The case vignettes analysed in this study provided rich insight into the challenges students experienced during their clerkship year beyond the domain of the “Medical Expert” role. “Challenging cases” occurred over all disciplines and the whole age spectrum with an emphasis on emergencies and emotionally touching situations. Underlying themes mostly related to communication and issues of professionalism. Our findings should encourage clinical supervisors to explicitly address these highly relevant issues during workplace training.

## List of abbreviations

- CanMEDS: Canadian Medical Education Directives for Specialists
- CCY: Clinical Clerkship Year
- OSCE: Objective Structured Clinical Examination
- SCLO: Swiss catalogue of learning Objectives

## Ethics approval

All analyzed data were part of the routine course assessment. Any information, which could have identified individuals or institutions was anonymized prior to analysis. According to the ethical committee of the Canton of Zurich formal consent by the students was not necessary. The committee stated no objections to the study (No. 099-2015).

## Authors' contributions

**PB** contributed to the study design, collection of data, further analyzed and interpreted the data including statistics, and prepared the first draft of the manuscript and contributed to finalization of the manuscript. **CM-F** contributed to study design and interpretation of data, participated in the ratings of case vignettes and contributed to the final version of the manuscript. **AWB** contributed to study design and interpretation of data, participated in the ratings of case vignettes and contributed to the final version of the manuscript. **JB** designed the study, contributed to data collection, participated in rating of case vignettes and finalized the manuscript. 

## Competing interests

The authors declare that they have no competing interests. 

## Supplementary Material

Assignment for students to select a “difficult clinical case” (translation from German).

Matrix used for evaluation of "difficult clinical cases"

Age distribution of patients (in years)

Comparison of the weighed frequencies of CanMEDS roles (top) and the roles assigned with first priority (mean of all three raters) (bottom).

Most frequent triple combinations of CanMEDS roles.

## Figures and Tables

**Table 1:**
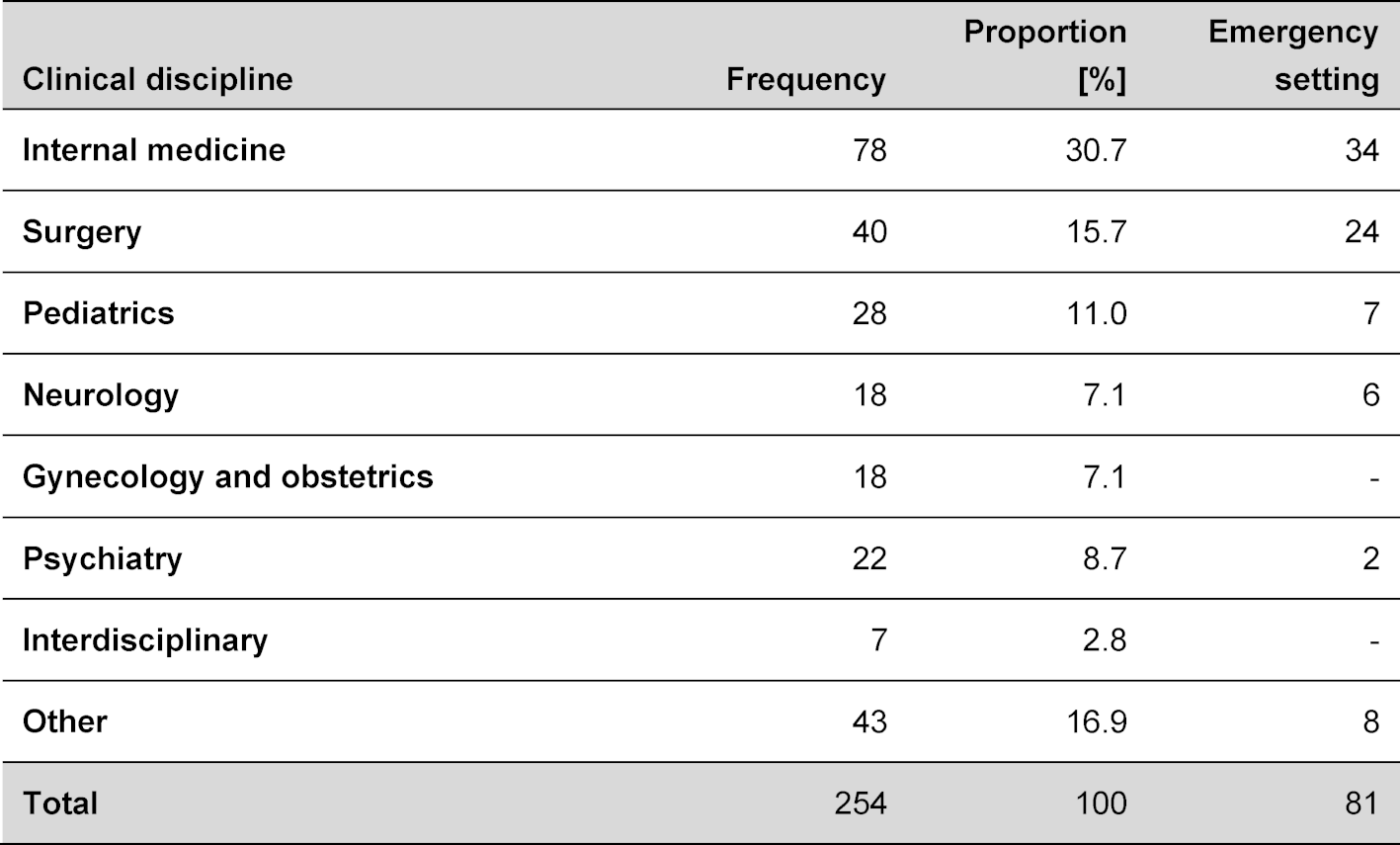
Distribution of clinical disciplines and underlying diseases

**Table 2:**
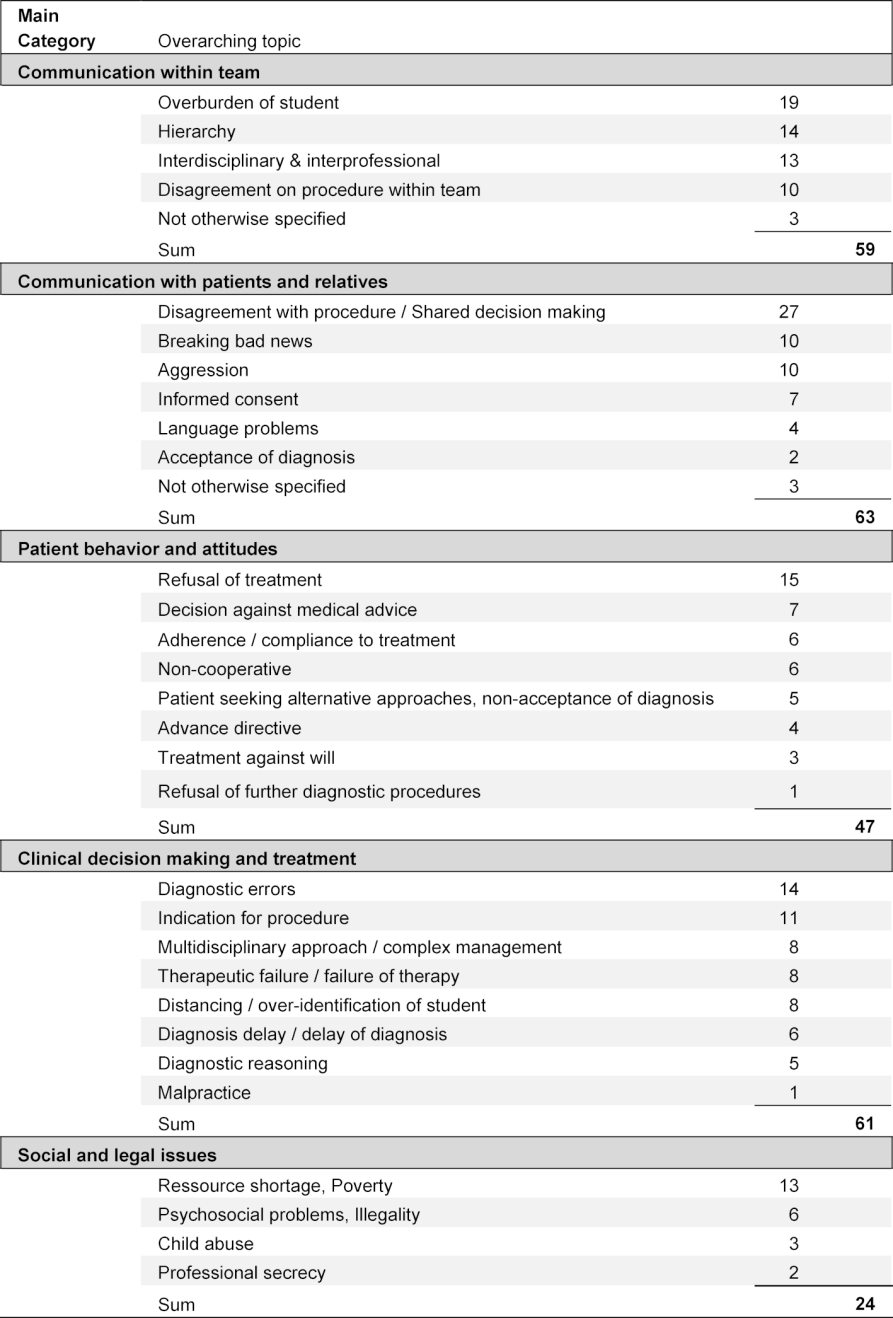
Overview of themes emerging from case analyses (n=254)
