# Supplementary material for: Challenging cases during clinical clerkships beyond the domain of the “medical expert”: an analysis of students' case vignettes
Source: GMS J Med Educ. 2019 May 16;36(3):Doc30. doi: 10.3205/zma001238 (PMC6545608; doi:10.3205/zma001238)
Supplement: Assignment for students to select a “difficult clinical case” (translation from German). [file JME-36-3-30-s-001.pdf]

Think of a situation from your clinical elective year, which was difficult to find a solution, e.g. because medical algorithms had not been sufficient for problem solving, or certain principles of treatment had contradicted each other, or persons had come into conflict with each other, or another problematic situation in patient care had occurred.

To arrive at an appropriate case, following questions might be helpful:

- *did any case occur, which I would have liked to discuss with one of my supervisors, but there was no opportunity for that ?*
- *did I experience a role conflict (including an internal conflict for my own person) ?*
- *a case where I could not find sleep about ?*
- *does anything still make your fingers itch ?*

Please, write a short text in note form of a quarter to half of a page, including

(a) treatment setting (outpatient, inpatient, emergency, etc.)
(b) age, gender, occupation of patients
(c) main diagnosis
(d) problem of the case in respect to the upcoming workshop
(e) was the described problem solved - and if so, how?
