# Supplementary material for: Challenging cases during clinical clerkships beyond the domain of the “medical expert”: an analysis of students' case vignettes
Source: GMS J Med Educ. 2019 May 16;36(3):Doc30. doi: 10.3205/zma001238 (PMC6545608; doi:10.3205/zma001238)
Supplement: Matrix used for evaluation of "difficult clinical cases" [file JME-36-3-30-s-002.pdf]

Assessment matrix: difficult clinical case					Case No.	
Age		Department				
Sex		Disease				
	Theme					
	(E.g.: end-of-life; breaking bad news; team-conflict; hierarchy-conflict; pat-autonomy; deportation; treatment of relatives; clinical decision dilemma; legal uncertainty)					
	Problem of Migration?			Palliative Care?		

CanMEDS-Role 1		Professional	Communicator
Role 2		Scholar	Collaborator
Role 3		Health Advocate	Manager
pro / com / coll / man / HA / scho / medEx		Medical Expert	
