# Supplementary figures and images for: Challenging cases during clinical clerkships beyond the domain of the “medical expert”: an analysis of students' case vignettes
Source: GMS J Med Educ. 2019 May 16;36(3):Doc30. doi: 10.3205/zma001238 (PMC6545608; doi:10.3205/zma001238)

## Age distribution

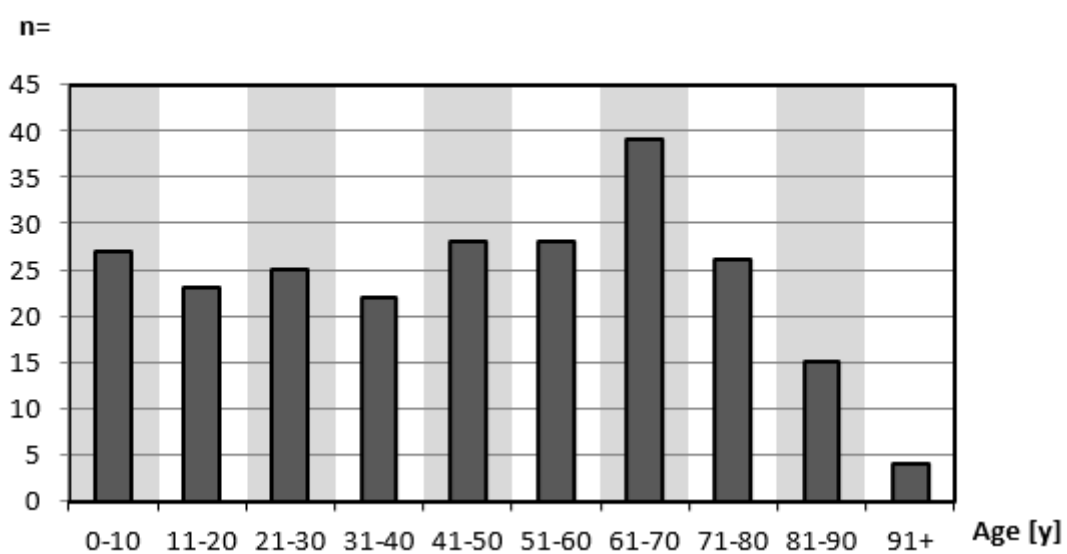

Supplement: Age distribution of patients (in years) [file JME-36-3-30-s-003.pdf]
