# Supplementary material for: Challenging cases during clinical clerkships beyond the domain of the “medical expert”: an analysis of students' case vignettes
Source: GMS J Med Educ. 2019 May 16;36(3):Doc30. doi: 10.3205/zma001238 (PMC6545608; doi:10.3205/zma001238)
Supplement: Comparison of the weighed frequencies of CanMEDS roles (top) and the roles assigned with first priority (mean of all three raters) (bottom). [file JME-36-3-30-s-004.pdf]

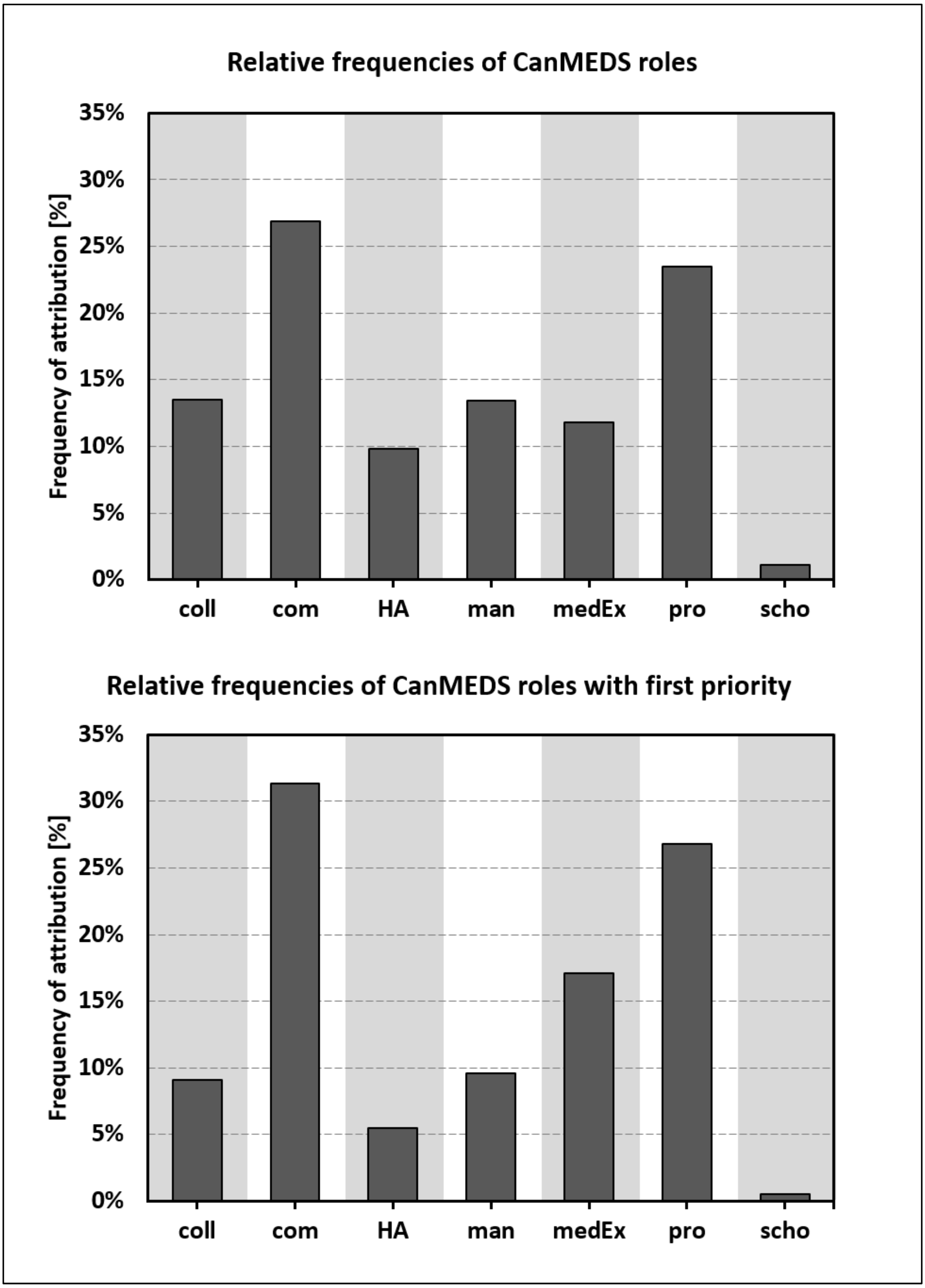
Relative frequencies of CanMEDS roles
Frequency of attribution [%]
35%
30%
25%
20%
15%
10%
5%
0%
coll
com
HA
man
medEx
pro
scho
Relative frequencies of CanMEDS roles with first priority
Frequency of attribution [%]
35%
30%
25%
20%
15%
10%
5%
0%
coll
com
HA
man
medEx
pro
scho
