# Supplementary material for: Challenging cases during clinical clerkships beyond the domain of the “medical expert”: an analysis of students' case vignettes
Source: GMS J Med Educ. 2019 May 16;36(3):Doc30. doi: 10.3205/zma001238 (PMC6545608; doi:10.3205/zma001238)
Supplement: Most frequent triple combinations of CanMEDS roles. [file JME-36-3-30-s-005.pdf]

Combinations of roles			Frequency (n)
Communicator	Professional	Manager	68
Communicator	Professional	Health Advocate	67
Communicator	Professional	Collaborator	61
Communicator	Manager	Collaborator	54
Professional	Manager	Collaborator	48
Professional	Manager	Health Advocate	32
Communicator	Manager	Health Advocate	27
Communicator	Health Advocate	Medical Expert	26
Communicator	Professional	Medical Expert	24
Professional	Health Advocate	Scholar	22

(n=sum of the combinations chosen by the raters, Most frequently assigned roles highlighted grey)
